# Feasibility and initial outcomes of a multifaceted prevention programme of melioidosis in diabetic patients in Ubon Ratchathani, northeast Thailand

**DOI:** 10.1371/journal.pntd.0006765

**Published:** 2018-09-06

**Authors:** Pornpan Suntornsut, Wipada Chaowagul, Wilasinee Thongklang, Thidarat Phosri, Nicholas P. J. Day, Susan Michie, Direk Limmathurotsakul

**Affiliations:** 1 Mahidol-Oxford Tropical Medicine Research Unit, Faculty of Tropical Medicine, Mahidol University, Bangkok, Thailand; 2 Department of Medicine, Sunpasitthiprasong Hospital, Ubon Ratchathani, Thailand; 3 Centre for Tropical Medicine and Global Health, Nuffield Department of Medicine, University of Oxford, United Kingdom, United Kingdom; 4 Centre for Behaviour Change and Department of Clinical, Educational and Health Psychology, University College London, London, United Kingdom; 5 Department of Tropical Hygiene, Faculty of Tropical Medicine, Mahidol University, Bangkok, Thailand; Charles Darwin University, AUSTRALIA

## Abstract

**Background:**

Melioidosis is an infection caused by *Burkholderia pseudomallei*, a Gram-negative bacillus found in soil and water. Diabetes mellitus is the most important risk factor for melioidosis. The recommendations for disease prevention include avoiding direct contact with soil and water, and drinking only boiled or bottled water.

**Methods:**

A prospective intervention study was conducted to evaluate the feasibility and behavioural outcomes of a multifaceted prevention programme for melioidosis. Participants were diabetic adults in Ubon Ratchathani, northeast Thailand. Ten behavioural support groups consisting of 6 to 10 participants per group were conducted. Twelve behaviour change techniques were used: information about health consequences, credible source, adding objects to the environment, reconstructing the physical environment, instruction on how to perform a behaviour, demonstration of the behaviour, commitment, prompts/cues, self-monitoring of behaviour, goal setting, feedback on behaviour, and social support, and their feasibilities evaluated.

**Results:**

There were 70 participants, of median age 59 years and 52 (74%) were female. Participants found the intervention beneficial, interesting and engaging. Participants indicated that they liked to watch videos with information about melioidosis delivered by local doctors and patients who survived melioidosis, and videos showing use of over-the-knee boots by local farmers. Participants felt engaged in the sessions that trialed protective gear and that made calendars with individual photographs and self-pledges as a reminder tool. The proportions of participants reporting that they always wore boots while working in rice fields increased from 30% (10/33) to 77% (28/37, p = 0.04), and that they drank only boiled or bottle water increased from 43% (30/70) to 86% (59/69, p<0.001) at 6 months post intervention.

**Conclusion:**

The programme is highly acceptable to participants, and can support behaviour change. Policy makers should consider implementing the programme in areas where melioidosis is endemic. Making calendars with individual photographs and self-pledges as a reminder tool could be powerful in behaviour change interventions, and further research on this component is needed.

## Author summary

Melioidosis is a serious infectious disease caused by the Gram-negative environmental bacterium, *Burkholderia pseudomallei*. Infection in humans occurs following skin inoculation, inhalation or ingestion. The recommendations for melioidosis prevention include using protective gear such as rubber boots when in direct contact with soil and environmental water, and drinking only boiled or bottled water. A multifaceted prevention programme is recommended to achieve the desired behaviour changes. Here, we evaluated the feasibility and behavioural outcomes of a multifaceted prevention programme for melioidosis. Our study participants were diabetic adults in Ubon Ratchathani, northeast Thailand. We found that the multifaceted prevention programme was highly acceptable to participants, and could support behaviour change. A calendar with an individual photograph as a reminder tool engaged participants effectively. Our study also confirmed that commitment and action by the government are essential for the preventive interventions to be successful. We recommend that policy makers should consider implementing the programme in areas where melioidosis is endemic. Since cultures and barriers to adopting the recommended behaviours vary, the intervention strategies would need to be adapted to local contexts.

## Introduction

Melioidosis is an often fatal infectious disease caused by a Gram-negative bacterium, *Burkholderia pseudomallei*, which is commonly present in soil and water in tropical regions [1]. A spatial modeling study estimated that there are about 165,000 human melioidosis cases per year worldwide, of which 89,000 (54%) die [2]. The disease is highly endemic and commonly reported in Southeast Asia and northern Australia, where the mortality from melioidosis is about 40% and 14%, respectively [3, 4]. Melioidosis occurs through ingestion, inoculation, or inhalation of the bacterium through direct contact with an environmental soil or surface water [5]. Diabetes mellitus is the most important predisposing factor for melioidosis, and is present in about half of all melioidosis patients [6]. Therefore, persons with diabetes are the main targets for disease prevention interventions. No melioidosis vaccine is currently available [7]. Activities associated with an increased risk of disease acquisition in Thailand, where disease is highly endemic, include working in a rice field, other activities associated with exposure to soil or water, and drinking untreated water [5]. The recommendations for disease prevention include using protective gear such as rubber boots when in direct contact with soil and environmental water, and consuming only boiled or bottled water [5]. However, only a small proportion of people follow such recommendations [8].

Lack of adoption of these preventive behaviours still occurs in Thailand even though the Ministry of Public Health (MoPH) has been recommending wearing rubber boots and drinking boiled water, and provides free rubber boots to prevent leptospirosis since a rise in leptospirosis incidence in 1996 [9, 10]. In a previous focus group study, we identified barriers to adopting recommended preventive behaviours in Thailand [8]. The main barriers were categorized into five domains: (i) knowledge, (ii) beliefs about consequences, (iii) intention and goals, (iv) environmental context and resources, and (v) social influence. People have little knowledge of melioidosis, believe that there is little or no harm in not adopting the recommended preventive behaviours, and are not inclined to use boots while working in muddy rice fields [8, 11]. People perceived rubber boots to be hot and uncomfortable, and they normally followed the behaviour of friends, family and their community, the majority of whom did not wear boots while working in rice fields and did not boil water before drinking [8].

To change behaviour, interventions based on the factors that influence adherence to recommendations are needed [12–14]. In general, providing information and protective gear alone do not change their behaviour [8]. Two related frameworks have been developed to support the investigation of a wide range of possible influences on behaviour: the Theoretical Domains Framework (TDF), and the Behaviour Change Wheel (BCW) [12–14]. The TDF is a useful framework for understanding the barriers and factors influencing specific behaviours [12, 13, 15, 16], while the BCW is a comprehensive framework that links this understanding to designing interventions including the recommended behaviour change techniques (BCTs) [14, 17]. Using these frameworks, we previously selected recommended behaviours, defined barriers to adopting those recommended behaviours, identified intervention options and modes of delivery, and developed a multifaceted prevention programme including a set of BCTs aimed at changing behaviours to prevent melioidosis, based on the local context in Thailand [8].

In this study, our aim was to evaluate the feasibility and behavioural outcomes of this multifaceted prevention programme for melioidosis [8] in diabetic adults in Ubon Ratchathani, northeast Thailand.

## Methods

### Study design

We conducted a study of a multifaceted prevention programme for melioidosis between April and December 2015. This was a small group intervention, in which 6 to 10 participants at a time attended a behavioural support group conducted by the study team. Each session lasted about 50 to 60 minutes. The intervention was provided once. Participants were then followed up by phone after one, two, four and five months, and by visiting homes on months three and six after the intervention. Feasibility of the intervention was determined by direct observation during the intervention, and by questionnaires and individual interviews after the intervention and at each follow-up. Components of the intervention were modified after each session based on feedback about feasibility. Two recommended preventive behaviours, wearing protective gear while working in rice fields and boiling water before drinking, were assessed prior to the intervention and at every follow-up by questionnaires and individual interviews.

The study sample was drawn from diabetic patients being followed up at five Tambon Health Promoting Hospitals (THPHs) in Ubon Ratchathani province, northeast Thailand. This comprised Non Noi THPH, Pak Kud Whai THPH, Pak Nam THPH, Ban Kok THPH and Hua Ruea THPH. THPHs are the first level of public health facility in Thailand.

### Participants

All patients attending for diabetic follow-up who had a physician-confirmed diagnosis of diabetes mellitus, were oriented and could converse normally were invited on the day by the study team to participate. Those who had been diagnosed with melioidosis and had not completed oral eradicative treatment for melioidosis were not eligible to participate because, per standard of care, those patients would be being advised to adopt the preventive behaviours to reduce the risk of melioidosis re-infection [18, 19]. The sample size target was defined by practical and resource considerations as 60 to 100 participants attending 8 to 12 sessions. We ended the study with 70 participants having completed 10 sessions because there was no new feedback to modify the interventions further; saturation was reached. As we aimed to assess intervention and methods feasibility, not effectiveness in terms of either behavioural or clinical outcomes, we did not conduct power calculations.

### Interventions

The interventions included 12 of 13 BCTs recommended in a focus group study evaluating barriers and recommended interventions to prevention melioidosis, conducted in Ubon Ratchathani province, Northeast Thailand in 2012 [8]. The recommended 13 BCTs include information about health consequences (e.g. explaining that not wearing boots while working in rice fields and that drinking untreated water can lead to an often fatal infectious disease called melioidosis), credible source (e.g. a high status professional in the government giving a speech that emphasises the importance of melioidosis prevention), adding objects to the environment (e.g. providing baby powder and long socks to alleviate the problem of discomfort due to heat and humidity when wearing boots), reconstructing the physical environment, instruction on how to perform a behaviour, demonstration of the behaviour, commitment, prompts/cues, self-monitoring of behaviour, goal setting, feedback on behaviour, feedback on outcome(s) of behaviour and social support [8]. The examples of BCTs specific to the two recommended preventive behaviours, wearing protective gear while working in rice fields and boiling water before drinking, had been previously described [8]. The recommended BCT of ‘feedback on outcome(s) of behaviour’ was not used because the study had short study duration and, therefore, could not determine clinical outcome of acquiring melioidosis over the study period. In this study, the objective of the intervention was to increase the frequency of the two recommended preventive behaviours: wearing boots while working in rice fields and dinking boiled or bottled water. Boiling water before drinking, rather than buying bottled water, was the main recommendation among those who were drinking untreated water. Buying bottled water was not primarily recommended because it could be considered expensive and it was not consistent with the national recommendation of boiling water before drinking [9]. Filtering water before drinking was not recommended because filters were rarely maintained properly and *B*. *pseudomallei* had been detected in filtered water samples previously [8, 20].

**Fig 1:**
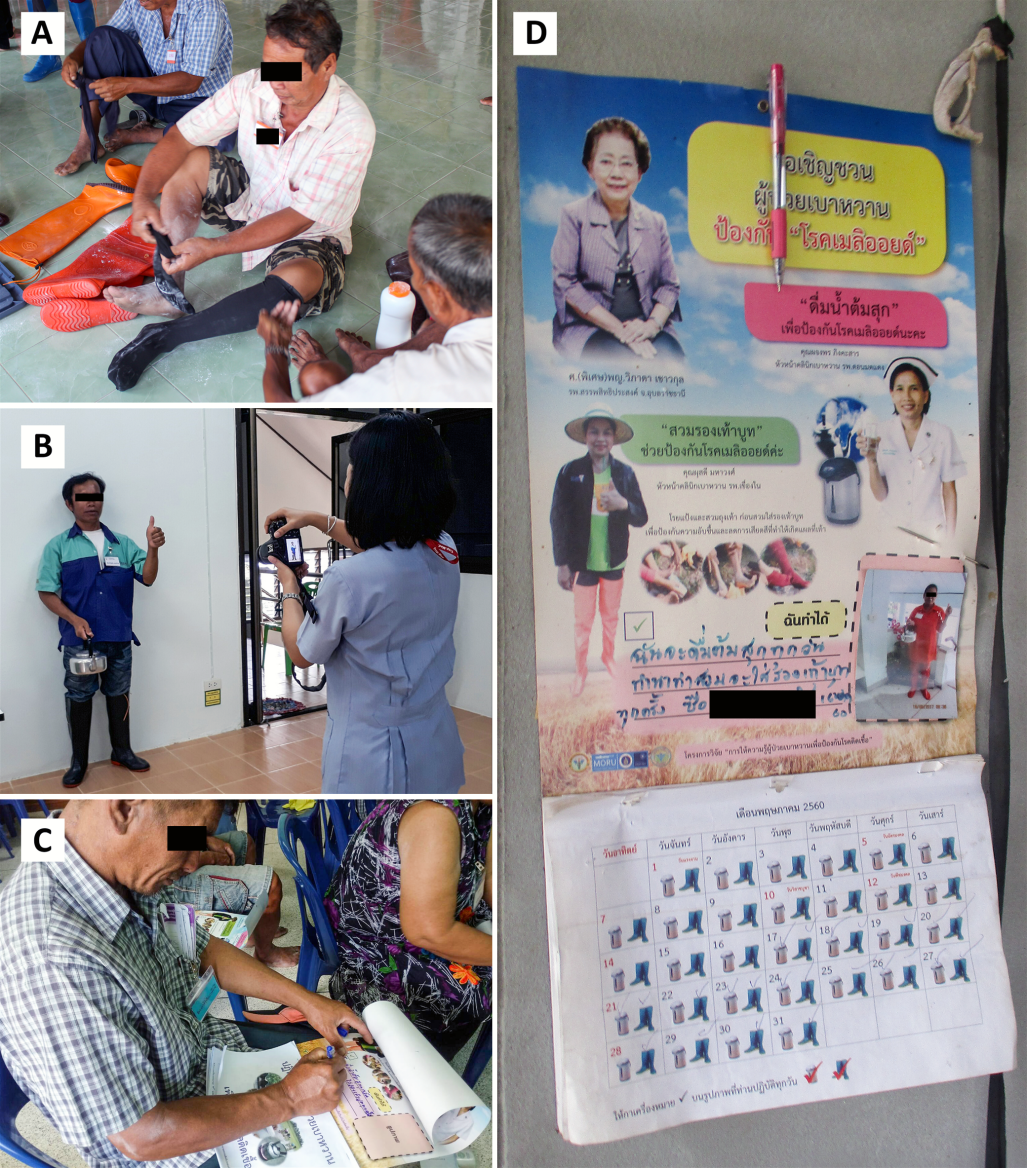
The sessions that trialed protective gear and that made calendars with individual photographs and self-pledge as a reminder tool. A. Multiple kinds of boots, long socks and baby powder provided for wear testing. B. The study team took photographs for each individual participant while wearing boots and holding a kettle. C The participant attached her individual photograph on the calendar and wrote her own pledge on the calendar. D. The calendar was hung in the participant’s house as a reminder tool.

The intervention package included six short videos, three pamphlets, and a calendar with a space for participants’ individual photographs and self-pledge. The materials are publicly available online (https://dx.doi.org/10.6084/m9.figshare.5734155). Each participant also received a pair of long socks and a bottle of baby powder (to reduce itching inside boots) and a 2-litre plastic ice bucket commonly-used to store water to drink while working in rice fields. In each behavioural support group, participants received an introduction by a moderator, watched each short video, and had short group discussions at the end of each video to foster autonomous motivation for the recommended preventive behaviours. Participants then had a protective gear trial session, in which multiple kinds of boots were provided for participants to test them out for wearing (Fig 1A). Next, the study team took a photograph of each individual participant while wearing boots and holding a kettle (Fig 1B) and printed photographs for each participant to use in the next session. Finally, participants attended a session to make their own calendar to act as a reminder tool for the recommended preventive behaviours. We asked participants to attach their individual photograph to the calendar and write their own pledge on the calendar by themselves (Fig 1C). It was recommended that the calendar be hung in participants’ houses (Fig 1D). The moderator also stimulated group discussion during, before and after the sessions. Additionally, we provided social support by giving information to nurses, doctors, participants’ relatives and health volunteers in each participating THPH about the intervention and potential benefits of the intervention. We also ask them to encourage the participant to continue with the recommended behaviours.

Components of the final programme, their related BCTs and intervention functions, and details of each short video are described in Table 1.

**Table 1:** Components of the multifaceted prevention programme of melioidosis and their related behaviour change techniques (BCTs) and intervention functions.

Components *	Recommended BCTs **	Intervention functions ***	Details of the activities
**Part I: about 35–40 minutes**			
Introduction and presenting three short videos. The videos were about melioidosis presented by infographics and healthcare officers (2.59 minutes), an interview with a family whose their relative died of melioidosis (0.59 minute) and an interview with three patients who survived acute melioidosis (2.02 minutes)	Information about health consequences and Credible source	Education, Persuasion	The videos explain about melioidosis, that the disease is highly endemic in the area, and that the disease is highly fatal. They explain that not wearing boots and gloves while working in rice fields and that drinking untreated water can lead to this disease. Credible source and persuasion reflect the fact that the videos are presented by healthcare officers, relatives and patients and also in local dialect. The moderator stimulated short group discussion at the end of each video to foster autonomous motivation for the recommended preventive behaviours.
Presenting a short video about how to boil water and restructure available objects to remind themselves to boil water and store boiled water for drinking (1.07 minute)	Restructuring the physical environment, instruction on how to perform a behaviuor, Demonstration of the behaviour, Credible source, Prompts/cues	Environmental restructuring, Education, Persuasion Modelling, and Enablement	The video explains that the causative bacteria can be present in drinking water, that boiling is the best method to kill those bacteria, that they could boil water after cooking so that it could be done routinely and that they should store boiled water so that they can have boiled water to drink at anytime they want. Credible source and persuasion reflect the fact that the video is presented by healthcare officers, in rural setting similar to participants’ houses, and also in local dialect.
Presenting two short videos about wearing boots. The former one was about different kinds of boots, and the benefit of over-the-knee boots to be used while working in rice fields (3.33 minutes) and the latter one was about wearing boots or shoes while walking or working in non-rice fields (0.59 minute)	Restructuring the physical environment, Instruction on how to perform a behaviour, Demonstration of the behaviour, Credible source, Prompts/cues	Environmental restructuring, Education, Persuasion Modelling, and Enablement	The first video explains that wellington boots, the commonly used boots, are not the only type of boots, and that wearing over-the-knee boots or waders allow walking in the flooded or muddy rice fields without difficulty. In addition, over-the-knee boots are durable enough to prevent cuts from golden apple snails, and wearing long socks and applying baby powder before wearing these boots could alleviate the problem of heat and humidity. The second video explains that the wellington boots can be used while walking or working in non-rice fields, and walking barefoot is not recommended. Credible source and persuasion reflect the fact that the videos are presented by healthcare officers and local people, in rice and non-rice fields, and also in local dialect.
Moderator asked participants to shout two-part phrases together and repeatedly (about 2 minutes) ****	Habit formation (Mental)	Education, Persuasion, Coercion	The moderator asked participants to repeat short two-part phrases. The moderator shouted the first part and asked the participants to shout the latter part together. The three phrases included (1) “to prevent–melioidosis”, (2) “work in rice fields–wear boots” (3) “drink–boiled water”. For each round, each phrase was repeated three times, and this BCT was conducted for two rounds.
**Part II: 15–20 minutes**			
Protective gear trial session (about 5 minutes)	Adding objects to the environment, Restructuring the physical environment	Environmental restructuring, Training, and Enablement	The moderator provided multiple kinds of boots, long socks and baby powder for wear testing.
Taking individual photographs	Identification of self as role model	Modelling and Persuasion	The study team took and print photograph for each individual participant while wearing boots and holding a kettle (Fig 1A and 1B)
Making their own calendar to act as a reminder tool	Prompts/cues, Credible source, Instruction on how to perform a behaviour, Demonstration of the behaviour, Identification of self as role model, Goal setting, Commitment and Social support	Modelling, Education, Persuasion, Environment restructuring, Enablement	The moderator asked participants to attach their individual photograph on the calendar and write a self-pledge on the calendar by themselves (Fig 1C). A list of recommended pledges for participants were provided.**** Participants could modify those for their own pledges. Examples of the pledge included, “I will always boil water before drinking” and “I will always wear boots while working in the rice fields.” The calendar also has the pictures of famous doctors and healthcare workers in the province, and pictures showing them wearing boots and drinking boiled water. The calendar had pictures of boots and a kettle on each date, and participants were asked to write a cross on those pictures when they performed the recommended behaviour on that day. This was to emphasise the behavioural goals of wearing boots 100% of the times working in rice fields and drinking only boiled water. The calendar was recommended to hang in participants’ houses (Fig 1D).
**Additional components**			
Informing families of the participants, healthcare workers, and community health volunteers about the prevention programme	Social support and Feedback on behaviour	Enablement, Education, Persuasion, and Coercion	The study team informed families of the participants, healthcare workers and community health volunteers about melioidosis, how to prevent melioidosis and the prevention programme. The study team asked the families of the participants and community heath volunteers to observe and inform the participant as to how often they wear boots and gloves while working in rice fields, and how often they drink boiled water

* All videos, including a video showing an example of mental habit formation used, are publicly available online (https://dx.doi.org/10.6084/m9.figshare.5734155).

** BCT is defined as an active component of an intervention designed to change behaviour. Recommended BCTs were identified by the behaviour change wheel (BCW) and APEASE criteria.

*** A BCT may have more than one function.

**** Implemented between the second and fourth group, and used since then.

### Measurements

#### Ethics statement

Approval for the study was obtained from the Institute for the Development of Human Research Protections, Ministry of Public Health, Thailand (ref 189/2557) and Oxford Tropical Research Ethics Committee, University of Oxford, United Kingdom (ref 06–14). Written informed consent was obtained from each participant prior to conducting each intervention group. Additional consent was obtained from each participant for photographs and video recordings, and for the records to be used for research, published reports of the work and educational materials.

#### Direct observation

In every session, one research assistant was assigned solely to observe the response of the participants. The observer coded variables related to the context of the intervention components and the activities in which participants were engaged. All sessions were scripted and video-recorded. The video and audio recordings and observation notes were reviewed by two authors (PT and DL) at the end of every session.

#### Questionnaires

The interviewee-based questionnaires were used at baseline (pre-intervention) and at monthly follow-ups. The variables included (1) “Did the participant work in rice fields or walked in muddy fields during last week?” (1.1) If yes, “Did the participant wear shoes or boots?”, with response options of “walked barefoot”, “wore flippers”, “wore boots sometimes”, “wore boots every time”, and “wore other shoes”, (2) “Did you drink water from any following sources?”, with response options of “well water”, “borehole water”, “pond water”, “rain water”, “tap water” and “bottled water”, (2.1) for any source of water, “Did you boil the water before drinking?”, with response options of “did not boil”, “boiled sometimes” and “boiled every time”.

Proportions of participants reporting that they wore boots every time while working in rice fields was defined as the number who answered that they “wore boots every time” compared with the number who answered that they “worked in rice fields or walked in muddy fields during last week”. Proportions of participants reporting that they drank only boiled or bottled water was defined as the number of those who answered that they “boiled every time” for all sources of water they drank except “bottled water” compared with the total number of participants.

#### Individual interviews

We interviewed each participant individually at the end of the intervention and at 3-month and 6-month follow-ups. We asked about the participants’ recall and evaluation of each component of the intervention, with response alternatives of “like”, “neutral” and “dislike”. We also asked the reason for their responses and whether they had any advice for improving each video, session procedure and materials. Each interview lasted about 30 minutes.

### Data analysis

Mixed methods were used to evaluate feasibility. Descriptive statistics presented interquartile ranges as 25^th^ and 75^th^ percentiles. Qualitative data from the direct observations during the intervention were analyzed using thematic analysis. McNemar’s exact test was used to compare the percentage of participants reporting that they performed recommended preventive behaviours before and after the intervention. McNemar’s test was used because the evaluation was a repeated measurement of the same subjects over time [21]. Statistical analyses were performed using Stata version 14.0 (StataCorp LP, College Station, TX).

## Results

### Participant characteristics

Of the 70 participants, 52 were female (74%) and the median age was 59 years old (interquartile range 52 to 65; range 32 to 77 years old). Forty-nine participants (70%) answered that they were farmers. Of 33 participants who worked in rice fields during the last week prior to the enrollment, six (18%) walked barefoot, 15 (45%) wore sandals, four (12%) wore boots sometimes and 10 (33%) wore boots every time while working in the rice fields. Boiling water from each source every time before drinking was reported in 2 of 4 participants (50%) who drank water from the well, 3 of 22 (14%) who drank borehole water, 4 of 14 (29%) who drank rainwater, and 8 of 29 (28%) who drank tap water. No participants drank water from a pond. Nine of 39 participants who drank bottled water (23%) also drank water from other sources without boiling. Overall, 30 (43%) drank only boiled or bottled water.

### Feasibility

During the first four sessions, we received many comments and advice from participants. Participants suggested that the duration of the videos should be shorter, and pointed to information that should be added to the videos or presented by local healthcare workers in the real local setting. Therefore, videos were revised and recut, and the median and maximum duration of the videos were reduced from 3 and 5 minutes to 1:30 and 3 minutes, respectively. The video showing that wearing boots can protect from being cut by golden apple snails was added. Videos showing how to boil water were remade and presented by local healthcare workers in the real local settings rather than presented by the study team in the urban setting.

Based on direct observation, we found that the script of the moderator to stimulate group discussion between each video was too long, and participants took a lot of time to come up with their own pledges. Therefore, the script was shortened and the study team made a list of common and recommended pledges for participants to see and modify for their own pledges. Examples of the pledge included, “I will always boil water before drinking” and “I will always wear boots while working in the rice fields”.

At the end of the first three group sessions, we found that many participants could not remember the name of the disease and the recommended behaviours. Therefore, in the fourth group, a BCT of mental habit formation was employed by asking participants to shout three short two-part phrases repeatedly. The moderator would shout the first part and then asked the participants to shout the latter part together. The three phrases included (1) “to prevent–melioidosis”, (2) “work in rice fields–wear boots” (3) “drink–boiled water”. For each round, each phrase was repeated for three times, and this BCT was conducted for two rounds. Feedbacks at the end of the fourth group session about this BCT were good, and all participants could remember the name of the disease and the two main preventive behaviours. This mental habit formation has been included as one of the main BCTs for the programme since the fourth group (Table 1).

The third session was longer than 60 minutes; after modifications in accordance with feedback, the last seven sessions were shorter than 60 minutes. We received no additional suggestions after the fourth session, and, therefore, no further changes were made from the fifth to the tenth session.

### Acceptability

Participants found the intervention beneficial, interesting and engaging. Features of the sessions that participants reported beneficial were the information about disease, and learning that applying baby powder and long socks could make wearing boots comfortable. Most participants had never heard of the disease and the consequences of the disease. Participants indicated that they liked to watch videos about melioidosis delivered by local doctors, relatives of those who died of melioidosis, and patients who survived melioidosis in the local dialect. Participants found that the video showing that farmers who wore over-the-knee boots could easily walk in muddy rice fields and that such boots were durable enough to protect themselves from golden apple snails. Many said that they had never known these things before.

Participants felt engaged in the sessions that trialed protective gear and that made calendars with individual photographs and a self-pledge as a reminder tool. Many participants reported that they saw over-the-knee boots available in the local market, but they had had no chance to try the boots and, therefore, had not known whether or not they would be comfortable and useful. We observed that most participants smiled while having their photos taken, holding their own photos, putting their photos on the calendar and writing their own pledges (Fig 1B and 1C). Based on the interactions and discussions between participants, our judgement was that most participants enjoyed the activities. During the home visits at the third and sixth month follow-ups, we found that 63/70 (90%) and 62/69 (90%) had their calendars hanging in the house, respectively. Many participants reported that they liked their own photos, as they had never had their own photo printed, and the picture of themselves wearing boots and holding a kettle was a good reminder tool for the recommended preventive behaviours. During the home visiting, we also observed that all participants had their boots at home, and many participants informed us that they had never used those boots until they attended our sessions. Most participants said that they would recommend attending the sessions to other diabetic patients.

### Behavioural outcomes

Sixty-nine participants completed the follow-up at 6 months after the intervention. One participant died of intracerebral hemorrhage 5 months after the intervention.

Proportions of participants reporting that they always wore boots while working in rice fields increased from 30% (10/33) to 74% (32/43) at 1-month post intervention (p<0.001). The proportion was stable at around 75 to 80%, and was at 76% (28/37) six months after the intervention (Table 2).

**Table 2:** Percentage of participants reporting that they performed preventive behaviours during the last week.

Time points	Always wearing boots while working in rice field	P value *	Drinking only boiled or bottled water	P value *
Prior to the intervention	30% (10/33)	-	43% (30/70)	-
1 month after the intervention	74% (32/43)	<0.001	81% (57/70)	<0.001
2 months after the intervention	73% (30/41)	<0.001	89% (62/70)	<0.001
3 months after the intervention	76% (28/37)	<0.001	83% (58/70)	<0.001
4 months after the intervention	85% (28/33)	0.002	83% (58/70)	<0.001
5 months after the intervention	79% (27/34)	0.004	81% (57/70)	<0.001
6 months after the intervention	76% (28/37)	0.04	86% (59/69) **	<0.001

* compared to baseline (prior to the intervention) and determined using McNemar’s exact test

** One participant died of cerebral hemorrhage prior to 6-month follow-up

The proportion of participants reporting that they drank only boiled or bottled water increased from 43% (30/70) to 86% (59/69) at 1-month post intervention (p<0.001). The proportion was stable at around 80 to 85%, and was at 86% (59/69) at 6-month after the intervention.

## Discussion

Our study shows that a multifaceted prevention programme for melioidosis is feasible and acceptable, and can prompt behaviour change in participants. Specifically, the proportion of participants wearing boots while working in rice fields and drinking only boiled or bottle water increased significantly after the intervention. Those increases were sustained for at least six months and are, therefore, likely to lead to lower risk of having melioidosis and other infectious diseases acquired via skin inoculation or ingestion [5]. These positive outcomes could be mainly because the programme was designed systematically based on the identified barriers and enablers, using the TDF and associated BCW [12–14] and taking into account the local context [8].

This may be the first study to show the efficacy of a calendar with an individual photograph as a reminder tool. The individual photograph of the participant wearing boots and holding a kettle could also be categorized as the BCTs “identification of self as role model” and “prompts/cue” [22]. Photography is a very powerful tool to convey a message [23–25]. Because our study could utilize boots and a kettle as part of the reminders, we used individual photographs with those objects. The photograph enables participants to understand the recommended behaviours. The combination of gestures, emotions, attitudes and facial expressions of participants in the photography allows participants to become directly engaged with the intervention. Devising individual photographs into a calendar could enhance the utility of the photographs as participants are likely to look at the calendar frequently, and feel more engaged with their photograph.

The intervention positively affected wearing boots and boiling water before drinking; however, a proportion of participants did not adopt the recommended behaviours. We found that the intervention could not remove all barriers. For example, over-the-knee boots could be used in flooded rice fields without causing difficulty in walking, but were still uncomfortable in hot weather. The BCTs ‘social support’ (including asking nurses, doctors, health volunteers, and families to encourage the person to continue with the recommended behaviours), and the BCT ‘credible source’ (including a high status professional in the government giving a speech emphasizing the importance of melioidosis prevention) in our small study had limited efficacy. This is because, during the follow-up, a number of participants who did not adopt the recommended behaviours did not believe in the ‘information of health consequence’ and noted that if the burden and mortality of melioidosis was real why had they never seen any information or campaign from the government via mass media, particularly on television.

Our study has several strengths. First, we showed that the intervention can lead to adopting recommended preventive behaviours in diabetic patients, who are a key target population for melioidosis prevention in Thailand [6, 7]. Second, the positive effect of the visual tool (a calendar with an individual photograph) to support the behaviour change is an innovation. In this study, the activity of making calendar as a reminder tool implements a number of BCTs; including prompts/cues, credible source, instruction on how to perform a behaviour, demonstration of the behaviour, identification of self as role model, goal setting, commitment and social support (Table 1). Although our study was not designed to evaluate efficacy of each BCT related to this activity, based on our interviews with participants during the home visits, most participants appeared to highly appreciate their own photography on the calendars. Due to strong positive feedback on this component of the intervention, further research should be conducted to evaluate the feasibility and utility of making a calendar with an individual photograph as a reminder tool for other behaviour changes across a range of settings.

The major limitation of this study is that long-term behaviour changes could not be measured and that the follow-ups may be part of the intervention as well as a method of evaluation as they could act as a reminder. Cost-efficacy analysis could not accurately be estimated from this feasibility study but is being evaluated in a subsequent large trial. Also, the programme may not be equally effective for all ages and socioeconomic groupings in the diabetic population in the whole country and beyond. It is possible that some barriers and cultures vary, and that the intervention strategies would need to be adjusted based on local context. Because the reliability of self-report cannot be assumed, we combined it with observation. Our interviews were done together with multiple home visits, during which we observed the boots and kettles that they said they regularly used. It is still possible that some participants may not report accurately, and further evaluating methods such as interviewing relatives and neighbours, and visits to rice fields without notice (but with prior consents from the participants) could be used in the future.

We recommend that health care providers together with policy makers in melioidosis-endemic areas should consider implementating multifaceted interventions for melioidosis prevention. Policy makers, health care providers and researchers should develop a working group to evaluate the feasibility of the interventions, adjust components of the interventions based on their own local context, and gradually implement the interventions. Policy makers should also focus on delivering disease education, particularly through mass media and implementing the multifaceted interventions through healthcare providers. Researchers should also evaluate the efficacy and effectiveness of the interventions which are gradually implemented.

### Conclusion

In this study, we evaluated the multifaceted prevention programme of melioidosis and found that the programme is feasible and could lead to adopting recommended preventive behaviours. We strongly suggest that commitment and action by the government are essential for the preventive programmes to occur and be successful. Making calendars with individual photographs and self-pledges as a reminder tool could be powerful in behaviour change interventions, and further research on this component is needed.
